# Probiotics and Antibiotics Use for the Prevention of Travelers’ Diarrhea Among Polish Tourists—Results from an Online Survey: Why Is Education Needed?

**DOI:** 10.3390/antibiotics14111064

**Published:** 2025-10-23

**Authors:** Martyna Biała, Michał Biały, Patrycja Leśnik

**Affiliations:** 1 Department of Infectious Diseases, Liver Diseases and Acquired Immune Deficiences, Faculty of Medicine, Wroclaw Medical University, 51-149 Wroclaw, Poland; 2 Department of Pediatric Dentistry and Preclinical Dentistry, Faculty of Medicine and Dentistry, Wroclaw Medical University, 50-425 Wroclaw, Poland; 3 Department of Microbiology, Faculty of Medicine, Wroclaw Medical University, 50-368 Wroclaw, Poland

**Keywords:** travel diarrhea, probiotics, antibiotics, prophylaxis

## Abstract

**Background/Objectives**: Traveler’s diarrhea (TD) is a pressing issue, affecting a significant portion of international travelers. Despite the guidelines discouraging routine use of antibiotics and the inconclusive evidence supporting probiotics for TD prevention, both remain popular prophylactic strategies. However, data on these practices among Polish tourists are currently lacking. The objective of this study was to assess the prevalence of probiotic and antibiotic use as preventive measures against TD among Polish travelers. **Methods**: A cross-sectional survey was conducted between July and August 2025 among adults with at least one international trip per year. An anonymous online questionnaire collected demographic data, travel frequency, and the use of probiotics and/or antibiotics for TD prophylaxis. **Results**: Of 873 respondents, 848 completed the questionnaire (median age: 35 years; 62.5% female). Probiotic use for TD prevention was reported by 24% of respondents (7.4% during all travels, 11.9% during trips outside of Europe, and 4.7% occasionally). Probiotic use was significantly associated with higher travel frequency, female sex, and younger age. Antibiotic use for TD prophylaxis was declared by 5.3% of participants, more common among those without comorbidities, with no significant association with age, sex, or travel frequency. Additionally, 21.6% of respondents reported regular, daily use of probiotics, though only 10.9% were recommended by physicians. **Conclusions**: This study’s findings reveal that a significant proportion of Polish travelers used probiotics for TD prevention, despite limited evidence and expert guidelines. A small subset of respondents used an antibiotic as TD prophylaxis. As global antibiotic resistance continues to rise, emphasizing antimicrobial stewardship in travel medicine practice is crucial. The fact that many individuals self-administer probiotics without medical consultation underscores the need for greater public education on the risks, benefits, and limitations of these interventions.

## 1. Introduction

Traveler’s diarrhea (TD) is the most common travel-related illness, affecting 30% to 70% of international travelers, depending on the destination and time of year [1]. While most cases resolve on their own, up to 10% of affected individuals require medical attention, and approximately 3% may need hospitalization [2]. TD is particularly prevalent in areas with inadequate sanitation, poor food handling practices, and limited access to clean water, resulting in significant health issues, economic burdens, and decreased travel quality. Tourists can reduce the risk of catching TD by practicing regular hand hygiene with soap and water or an alcohol-based hand disinfectant, drinking only bottled or boiled water, and avoiding ice cubes, raw, or undercooked foods. Despite its simplicity and promotion of food safety education, many people ignore these rules during travel. However, some food and drink safety factors are beyond tourists’ control (e.g., the temperature of food storage fridges, the best-before dates of products used in restaurants, and the hygiene of restaurants’ kitchens). There is no universal vaccine to prevent TD. Still, some vaccines provide additional protection against infectious agents transmitted by the fecal-oral route, e.g., vaccines against cholera, typhoid fever, and hepatitis A. Routinely using antibiotics as a preventive measure against TD is discouraged because of concerns about antimicrobial resistance and potential side effects. However, antibiotics may be warranted for short-term travelers who are high-risk hosts (e.g., immunocompromised individuals or patients with significant comorbidities) [1].

Probiotics, which are live microorganisms that provide health benefits when consumed in sufficient amounts, have emerged as a safer, non-pharmacological alternative aimed at maintaining a healthy balance of gut microbiota [3]. Though the clinical efficacy of probiotics is not straightforward, as it varies by strain, the current evidence is inconclusive. Both the International Society of Travel Medicine and the CDC Yellow Book have reported that there is insufficient data to support the routine use of probiotics for the prevention of TD [1,4]. Data showed that the global probiotics market size was priced at USD 87.70 billion in 2023 and is estimated to reach USD 220.14 billion by 2030 [5]. Although probiotics are frequently used to promote beneficial effects in healthy and ill patients, in vulnerable groups, probiotics may turn into opportunistic pathogens, causing life-threatening pneumonia, endocarditis, and sepsis [6,7,8]. Moreover, many probiotics are distributed as dietary supplements, which do not require the same rigorous quality control standards as medications [9]. This can lead to potential safety concerns and a risk of unnecessary harm. There is also limited data about potential harm associated with the long-term use of probiotics [9]. Despite the uncertainty regarding their benefits and the risk of rare but serious adverse events in vulnerable populations, an increasing number of travelers are administering probiotics as a preventive measure. Notably, there is a lack of data regarding this practice among Polish tourists. This study aims to address this gap by assessing the prevalence of probiotic and antibiotic use for the prevention of TD among Polish travelers.

## 2. Results

### 2.1. Demographic Data

A total of 873 tourists responded to the survey, and 848 of them fully completed the questionnaire and were included in the final analysis. Participants’ ages ranged from 20 to 69, with a median age of 35 years (Table 1). Most (n = 530, 62.5%) individuals were female.

### 2.2. Travel Details and Prophylaxis of TD

A third of travelers (32%) declared that they had undertaken a foreign tourist trip once a year, a significant part of respondents (62.1%) had traveled twice to four times a year, and 5.9% of individuals declared they had undertaken a foreign tourist trip more than four times a year. Analysis showed that 7.4% of participants had always used probiotics as a TD prophylaxis during all international travels, 11.9% of tourists declared that they had always used probiotics during trips outside of Europe, 4.7% of individuals answered that they had sometimes used probiotics during foreign travel, and 76% of tourists declared that they had never used probiotics as a TD prophylaxis (Table 2). Statistical analysis showed that tourists who declared at least two trips a year more frequently used probiotics as a TD prophylaxis compared with people who undertook a foreign trip once a year (*p* = 0.038). Women used probiotics as a preventive measure against TD more often than men (*p* = 0.047); moreover, younger tourists more frequently used probiotics compared with other individuals (*p* = 0.0001) (Table 3).

Comorbidities were reported by 29% of respondents. There was no statistical significance between the general occurrence of chronic diseases and probiotics use as a prophylaxis of TD; however, detailed analysis of comorbidities showed that people with thyroid diseases more often used probiotics as a preventive measure of TD than other individuals with chronic diseases (*p* = 0.031).

Taking antibiotics for TD prophylaxis was declared by 5.3% of all participants (Table 4). There was no statistical significance between antibiotic use as a TD prophylaxis and frequency of traveling (*p* = 0.0793). Moreover, statistical analysis showed no significance between sociodemographic characteristics (sex and age) and antibiotic use as a preventive measure of TD (*p* = 0.12, *p* = 0.86, respectively). People with no comorbidities more frequently use antibiotics as a TD prophylaxis compared with individuals with chronic diseases (*p* = 0.028) (Table 5).

Nearly 5.3% of all tourists answered that they used both probiotics and antibiotics as a TD prophylaxis at the same time.

A travel medicine specialist was consulted by 30% of respondents before the trip. Almost 91.1% of all tourists felt very well (20%) or rather well (71.1%) informed about the methods of TD prophylaxis. Nearly 14.2% of individuals felt rather poorly informed, and 0.7% of respondents declared that they felt definitely not informed about TD prophylaxis.

### 2.3. Daily Probiotics Use

Regular daily consumption of probiotics was declared by 21.6% of respondents, including 105 people (12.4%) who use probiotics for the improvement of digestive health and 78 individuals who use probiotics for a general health benefit (9.2%). Among these respondents, 25.1% declared that probiotics have been linked to improved health, 66.7% stated that probiotics have not been connected with a significant improvement in their health, and 8.2% of individuals have not reported any changes in health condition. The analysis showed that only 10.9% of probiotics were recommended to respondents by clinicians, and nearly 45.4% of probiotics were advised to participants by dietitians. Even 37.2% of individuals declared that they found online information about probiotics and decided to start daily consumption on their own. A total of 3.8% of respondents were encouraged to undertake daily probiotic use by pharmacists and 2.7% by family/friends, respectively.

## 3. Discussion

We found that 24% of all respondents reported using probiotics as a preventive measure against TD, with the following frequencies: 7.4% during all international travel, 11.9% during trips outside of Europe, and 4.7% sometimes during foreign travel. Moreover, people who declared at least two excursions a year more frequently used probiotics compared with individuals who undertook a foreign trip once a year. The systematic review and meta-analysis investigated the influence of probiotics on the prophylaxis of TD. They revealed that the prevalence of TD in individuals who used probiotics during travel ranged from 3.9% to 53.2%; in contrast, in control groups, the range was from 7.6% to 70.7% [10]. This analysis included randomized, double-blind, placebo-controlled trials involving adults aged ≥18 years with a travel history [10]. The literature search identified 10 different probiotics used by participants from analyzed studies, either as single strains or as a combination containing two or more different strains in dosages ranging from 2 × 10^8^ to 7 × 10^9^ CFU/d [10]. Travelers began using probiotics 1–5 days before their excursions and continued throughout their trip period. Data showed that several strains of probiotics, such as *L. acidophilus*, *L. rhamnosus*, *L. fermentum*, *S. cerevisiae*, and *S. boulardii*, displayed potential efficacy in diminishing the prevalence of TD [10]. However, the guidelines indicate that there is insufficient evidence to recommend probiotic use as a TD prophylaxis [1,4]. Further studies are needed to assess the proper probiotic strains, dosages, and length of probiotic courses, considering different travel destinations and heterogeneity of populations. Factors associated with the risk of developing TD include host factors (e.g., reduced stomach acid, comorbidities, younger age, and hand hygiene practices), travel destination (especially low- and middle-income countries), duration of trip (longer periods), style of travel (e.g., backpacking travels), and the oral and intestinal microbiota composition [11,12]. The study showed that a foreign trip considerably altered gut microbiota, resulting in reduced richness, decreased α-diversity, and a 40-fold increase in *Escherichia*/*Shigella* [12]. Moreover, travelers’ own microbiota can also influence the risk of TD. Mlangeni T et al. revealed that pre-travel gut microbial patterns enriched with *Ruminococcaceae* and *Coprococcus* were linked to an increased risk of acquiring diarrheagenic *E. coli*, and no specific pre-travel microbiota profile was identified as preventive against TD [12]. The inherent variability in a traveler’s own microbiome makes a universal approach to probiotics ineffective, and host and environmental factors lead to unique microbial communities with heterogeneous individual responses to probiotic strains. Recent data showed a significant role of the oral microbiome in preserving gut health and indicated that periodontal treatment might mitigate oral dysbiosis and alter gut microbial composition [13,14]. Dysbiosis may increase the risk of TD, and the mentioned data suggest that an imbalance in the oral cavity’s health might be potentially associated with a higher risk of TD.

Standard pre-travel advice to minimize the risk of TD often advises, “boil it, cook it, peel it, or forget it.” However, even tourists who follow these rules may still develop TD. Possible non-antimicrobial drugs for TD prophylaxis also include bismuth subsalicylate for children over 12 years and adults [1]. Nevertheless, the frequency of dosages (3 to 4 times daily) may decrease patient adherence and affect the efficacy of bismuth subsalicylate in the prevention of TD [15]. Fan H, et al. showed that bismuth subsalicylate, rifaximin, and probiotics were significantly associated with a diminished prevalence of TD when compared with the control group [16]. However, when this analysis considered the risk of bias and adverse event rate, rifaximin was more effective than probiotics and vaccines and had a better balance between benefit and harm compared with bismuth subsalicylate [16]. However, these data also revealed that bismuth subsalicylate was the most effective in participants who traveled to Mexico [16]. Antimicrobial prophylaxis for TD is recommended only in selective situations, especially in high-risk short-term travelers (immunocompromised individuals or tourists who have significant medical comorbidities) [1]. The global rise in resistance patterns limits the choice of antibiotics, and current guidelines discourage the use of doxycycline, TMP/SMX, or fluoroquinolones for TD prevention [1,17]. Alternative antibiotic options include rifaximin [1]. Prophylactic use of antibiotics does not protect against nonbacterial infections (viruses, protozoa). It may alter intestinal microbiota, increasing the risk for colonization of resistant bacterial pathogens, e.g., extended-spectrum beta-lactamase-producing *Enterobacteriaceae* [1,18,19]. Additionally, the use of antibiotics may be associated with side effects. Our study found that 5.3% of all respondents reported having used antibiotics as a preventive measure at some point, with frequencies of 0.2% during all international travel, 4.4% during trips outside of Europe, and 0.7% during some foreign travel. Moreover, individuals with no chronic diseases more frequently used antibiotic prophylaxis. These data showed that all tourists should be cautioned against unnecessary use of antibiotics, potential side effects, and risk of colonization with multidrug-resistant bacteria. 

We showed that 30% of respondents had a consultation with a travel medicine specialist before their trip. However, almost 91.1% of our respondents felt very well (20%) or at least well (71.1%) informed about the methods of TD prophylaxis. Nearly 14.2% of individuals felt rather poorly informed, and 0.7% of respondents declared that they thought they were definitely not informed about TD prophylaxis. AlAmer NA, et al. reported that 76.8% of participants had never sought pre-travel health consultations, and barriers to seeking travel medicine specialist consultation included perceived low risk (74.8%), lack of awareness (36.3%), lack of time (21.6%), and financial concerns (15.5%) [20]. The low rate of travel medicine consultations among travelers highlights the need for targeted educational campaigns about various health risks during excursions (e.g., risk of TD, malaria, dengue fever). 

The majority of previous studies focused on self-reported treatment of TD, not prophylaxis. To our knowledge, this is the first survey assessing the prevalence of probiotic and antibiotic use as a prophylaxis of TD among travelers. We observed a surprisingly high number of tourists who used probiotics as a preventive measure against TD, despite their effectiveness often being unproven or inconsistent, and a small subset of respondents who used unnecessary antibiotics as a prevention of TD. These results emphasize the need for education for travelers about TD prophylaxis. The expanding prevalence of antibiotic-resistant species highlights the importance of the rational use of antimicrobials among travelers. Estrada J, et al. showed that pre-travel medical consultation improved knowledge of travelers about TD [21]. However, 36.2% of them still believed that all tourists should use antibiotics to prevent TD, and 27.6% incorrectly indicated that any antibiotics taken during the excursion can be used to treat TD [21]. Another study revealed that carriage of standby antibiotics promoted less careful use of antibiotics [22]. Costello VH, et al. showed that antibiotic use for self-treatment was frequent in younger and older adults and often inappropriate, for example, for treatment of occasional loose stool, mild TD, and influenza-like symptoms [23]. These findings carry significant implications for medical counseling during pre-travel consultations and for preventing TD among tourists. Identifying gaps in tourists’ knowledge allows for the development of initiatives that enhance and promote awareness of preventive measures. Furthermore, regular courses on rational antibiotic prescribing are essential for pre-travel counseling medical doctors because of the need to diminish the risk of antimicrobial resistance. Study showed that empiric antibiotics for TD were prescribed in over 75% of pre-travel Global TravEpiNet consultations, but antimicrobial prescribing decreased steadily between 2009 and 2018, and fluoroquinolones were less often used than azithromycin, especially after the Food and Drug Administration warning about fluoroquinolones in 2016 [24]. Before antibiotic prescription, medical doctors should carefully assess the dangers of antibiotic overuse, side effects, and the risk of moderate-to-severe TD in areas lacking adequate medical services.

We also analyzed regular, daily probiotics use among respondents, and we found that 183 (21.6%) individuals declared daily consumption of probiotics, including 105 people (12.4%) who use probiotics as an improvement of digestive health, and 78 individuals who use probiotics as a general health benefit (9.2%). Among these respondents, 25.1% reported that probiotics have been linked to improved health, 66.7% stated that probiotics have not been connected with significant improvements in their health, and 8.2% of individuals have not reported any changes in their health condition. Moreover, our analysis showed that only 10.9% of probiotics were recommended to respondents by clinicians, and nearly 45.4% of probiotics were advised to participants by dietitians. A total of 37.2% of individuals reported finding online information about probiotics and deciding to consume them daily on their own. 3.8% of respondents were encouraged to use probiotics daily by pharmacists, and 2.7% by family/friends, respectively. Nowadays, a significant percentage of adults are turning to herbs, supplements, and probiotics to support their health. This surge is associated with a rising awareness of a healthy lifestyle, interest in preventive healthcare, many observational studies regarding the benefits of these products, and consumers’ belief that probiotics and other supplements work. However, the inherent variability of a person’s own microbiome makes a universal approach to probiotics ineffective. Probiotics are generally safe for most individuals. Still, in patients with severe comorbidities/weak immune systems, they may cause a range of serious side effects such as sepsis, endocarditis, and pneumonia [25,26,27,28,29]. Xu R, et al. conducted a literature review about risks associated with probiotics, and they divided them into two groups: intrinsic risks, including production of toxins and invasive factors, emboli, biofilm formation, antibiotic resistance with relevant genetic materials, genetic plasticity, mutation, and horizontal gene transfer, metabolic complications and extrinsic risks including problems in regulatory strength and public awareness (e.g., categorization of probiotics, evidence of safety, risk of contamination, lack of uniform and clear regulatory), host health status (people with immunodeficiency, critically ill patients, newborns), and appropriate administration [30]. Moreover, there is limited data about long-term concerns associated with probiotics use. Researchers suggested that future efforts should focus on developing tailored probiotics based on individual genetic makeup, own microbiome composition, and coexisting health conditions [30]. Based on this, probiotics are not universal “super pills,” and their effectiveness is often unproven or inconsistent, especially for people with comorbidities. Our study also indicated that a significant percentage of respondents usually use probiotics without consulting medical doctors because they are widely available as supplements. More public awareness and education about the use of probiotics as a preventive measure for TD, along with their regular daily consumption, are needed because of the significant gaps in understanding of their efficacy, safety, and appropriate use.

Limitations: Our study may not be representative of the whole population of Polish tourists. We did not analyze strains of probiotics, the duration of prophylaxis of TD, or travel destinations. We also did not investigate the prevalence of TD reported by respondents. Moreover, this study relies on online survey data, which may introduce recall and selection bias.

## 4. Materials and Methods

A cross-sectional study was conducted between July and August 2025. The anonymous survey was prepared as an online questionnaire directed at individuals who declared traveling abroad. This study was voluntary, and inclusion criteria included: age ≥ 18 years, travel history (at least one international trip a year), and Polish citizenship. Information about the questionnaire was disseminated through both classical channels (oral and written invitations) and online channels (e-mails, social media), aiming to encourage travelers to participate in the survey. Before participating in the survey, individuals were informed about the objectives of the study, anonymity, voluntary participation, and their right to withdraw from the study at any time. The questionnaire was divided into two sections: one for demographic data, travel details, and prophylaxis information, and another for daily probiotics use. Demographic data included: age and sex (male, female, or prefer not to say). Traveling details were concentrated on the frequency of international trips per year, use of probiotics and/or antibiotics as a TD prophylaxis, and frequency of a pre-travel consultation with a specialist. We also analyzed participants’ comorbidities.

### 4.1. Statistical Analysis

Statistical analyses were used to explore relationships between demographic factors (age, sex) and travel details, including probiotic and antibiotic use. Descriptive statistics were employed to sum up the distribution of participants by sex (male/female/other) and age groups (group 1: 20–30 years old, group 2: 31–40 years old, group 3: 41–50 years old, group 4: over 50 years old). A chi-squared test was used to compare qualitative variables. All statistical analyses were performed using Statistica 13.3, and a *p*-value of <0.05 was assumed statistically significant.

### 4.2. Ethical Approval

This study was conducted in accordance with the Declaration of Helsinki. The opinion of the Bioethical Committee of Wroclaw Medical University was obtained (positive opinion no. KB 299/2025).

## 5. Conclusions

Our study demonstrated a moderate prevalence of probiotics use as a preventive measure against TD among Polish tourists. Moreover, regular, daily consumption of probiotics was declared by about one-fifth of respondents. Only a small subset of respondents had used an antibiotic as a TD prophylaxis, but most of them had no comorbidities. These findings underscore the importance of education in preventive measures for TD, as many travelers do not adhere to current recommendations. Detailed guidance containing information on how to reduce the risk of TD and when to use antimicrobials as a TD prevention—readily accessible during travel—should be offered during the pre-travel consultation. As global antibiotic resistance continues to rise, emphasizing antimicrobial stewardship in travel medicine practice is crucial. Moreover, public awareness campaigns about the benefits and harms of probiotics use are needed. Further studies are needed to assess travel-related risk behaviors and preventive measures of TD among tourists, as well as antibiotic prescription practices at the pre-travel consultation.

## Figures and Tables

**Table 1:** Age of participants.

	n	Average	Median	Minimum	Maximum	25Q	75Q	SD
Age [years]	848	36.3	35.0	20.0	69.0	29.0	41.0	9.4

**Table 2:** Taking probiotics for TD prophylaxis among Polish tourists.

Percentage of Respondents	Frequency of Using Probiotics as a Preventive Measure of TD
7.4% (n = 63)	During all international travels
11.9% (n = 101)	During trips outside of Europe
4.7% (n = 40)	Sometimes
76.0% (n = 644)	Never

**Table 3:** Comparison of sociodemographic characteristics and comorbidities of participants according to probiotics use.

Variables	Probiotic Use (%)	*p*
Sex:		0.047
- Female 530 (62.5%)	164/530 (30.9%)	
- Male 318 (37.5%)	40/318 (12.7%)	
Age:		0.0001
- 20–30 years old: 246	87/246 (35.4%)	
- 31–40 years old: 370	83/370 (22.4%)	
- 41–50 years old: 164	29/164 (17.7%)	
- over 50 years old: 68	5/68 (7.4%)	
Comorbidities:		0.283
Yes: 246, including:	53/246 (21.5%)	
(a) hypertension: 106	6/106 (5.7%)	
(b) thyroid diseases: 47	20/47 (42.6)	
(c) atopic dermatitis: 6	2/6 (33.3%)	
(d) hypercholesterolemia: 30	7/30 (23.3%)	
(e) depression: 2	1/2 (50%)	
(f) diabetes: 53	16/53 (30.19%)	
(g) liver cirrhosis: 2	1/2 (50%)	
No: 602	151/602 (25.1%)	

**Table 4:** Taking antibiotics for TD prophylaxis among Polish tourists.

Percentage of Respondents	Frequency of Using Antibiotics as a Preventive Measure of TD
0.2% (n = 2)	During all international travels
4.4% (n = 37)	During trips outside of Europe
0.7% (n = 6)	Sometimes
94.7% (n = 803)	Never

**Table 5:** Comparison of sociodemographic characteristics and comorbidities of participants according to antibiotic use.

Variables	Antibiotic Use (%)	*p*
Sex:		0.12
- Female 530 (62.5%)	23/530 (4.3%)	
- Male 318 (37.5%)	22/318 (6.7%)	
Age:		0.86
- 20–30 years old: 246	15/246 (6.1%)	
- 31–40 years old: 370	17/370 (4.6%)	
- 41–50 years old: 164	9/164 (5.5%)	
- over 50 years old: 68	4/68 (5.8%)	
Comorbidities:		0.028
Yes: 246, including:	5/246 (2.0%)	
(h) hypertension: 106	1/106 (0.94%)	
(i) thyroid diseases: 47	2/47 (4.26%)	
(j) atopic dermatitis: 6	0/6 (0.0%)	
(k) hypercholesterolemia: 30	1/30 (3.33%)	
(l) depression: 2	0/2 (0.0%)	
(m) diabetes: 53	2/53 (3.77%)	
(n) liver cirrhosis: 2	0/2 (0.0%)	
No: 602	40/602 (6.6%)	

## Data Availability

Data presented in this study are available and can be shared upon reasonable request sent to the corresponding author.
